# Development of a Deep-Learning-Based Computerized Scoring Algorithm

**DOI:** 10.3390/s25082537

**Published:** 2025-04-17

**Authors:** Junghyun Heo, Layoung Hwang

**Affiliations:** 1 Department of AI Design, College of Design, Kookmin University, Seoul 02707, Republic of Korea; jh.heo@kookmin.ac.kr; 2 Department of Digital Healthcare, Yonsei University, Wonju 26493, Republic of Korea

**Keywords:** polygraph, lie detection, computerized scoring algorithm, bio-signals, deep learning, deep neural network

## Abstract

During polygraph tests, the examiner evaluates physiological responses recorded on a chart to identify deception. Generally, this evaluation involves a numerical scoring system. However, biases related to politics, region, and religion, as well as personal factors such as fatigue and stress, can lead to inaccuracies in the examiner’s judgment. To solve these problems, computerized scoring systems (CSSs) that automatically analyze charts have been introduced, aiming to reduce human error. Conventional CSS models, which rely on linear classifiers, struggle with the nonlinear nature of biological signals, resulting in poor performance. Therefore, it is crucial to incorporate deep learning structures such as deep neural networks, which account for the nonlinearity of bio-signals, to enhance effectiveness of CSSs. This paper introduces a Korean computerized scoring system that leverages a deep neural network, which was developed to mitigate the subjective bias of polygraph examiners and to obtain high-accuracy results by considering the nonlinearity of bio-signals. The performance of the developed algorithm was evaluated, demonstrating recall, precision, and F1 scores of 0.9681 ± 0.0314, 0.9700 ± 0.0321, and 0.9683 ± 0.0171, respectively. These results suggested a significant improvement in CSS performance over conventional systems that depend on linear classifiers.

## 1. Introduction and Literature Review

### 1.1. Polygraph Test

A polygraph visually tracks physiological changes in an individual as they answer specific questions. Analog polygraphs use ink pens on paper; modern ones use software to record digital data directly to computers.

Exposure to lies triggers the sympathetic nervous system arousal during polygraph testing, causing physiological changes detected through blood pressure, respiration, and skin conductance measurements. Monitoring includes cuff blood pressure readings, respiratory bellow monitoring via a cuff-type sphygmomanometer, and skin conductance assessment via finger electrodes, all simultaneously recorded [1,2].

The polygraph examination is an intricate analysis of physiological responses aimed at detecting deception. Each test comprises 10–12 questions, spanning a total duration of 5 min. Only questions pre-reviewed with the polygraph examiner during the pre-test interview are allowed, and the participant can only respond with “yes” or “no”. The primary techniques used in the polygraph test include the comparison question test (CQT) and the concealed information test (CIT) [3,4].

### 1.2. CQT

The CQT assesses whether a participant has direct knowledge or experience related to the incident in question based on their responses. Predominantly, the CQT and its variations, such as the Utah and Backster techniques, are utilized in polygraph testing. The CQT employs three types of questions: irrelevant, relevant, and comparison. Irrelevant questions, which are neutral and unrelated to the incident, serve to monitor the participant’s baseline response and reduce tension. Relevant questions directly pertain to the incident under investigation. Comparison questions, while not directly related to the incident, bear similarities to it and are used to control the psychological state of the participant for a comparison with their response to relevant questions.

The premise of the CQT lies in the assumption that deceptive individuals exhibit higher physiological responses to relevant questions than to comparison questions, whereas truthful individuals show higher or equal responses to comparison questions than to relevant questions. The technique hinges on the existence of a response to a relevant question and the variance in responses to comparison and relevant questions to detect deception. This approach is grounded in the observation that both truthful and deceptive individuals exhibit significant responses to relevant questions over irrelevant ones. The polygraph examiner assigns a numerical score to the differences in responses to these questions to detect deception.

### 1.3. Numerical Scoring System

In the polygraph test, deception is detected by the examiner through the analysis of charts using a numerical scoring system (NSS). This system, initially popularized by Backster’s 7-point scoring method, has undergone enhancements through empirical research [5]. Presently, three modified models based on the 7-point system are predominantly used. These models, developed by the US Government [6], ASTM International [7], and Handler and Nelson [8], incorporate both the 7-point and 3-point scoring systems. The 3-point system is favored for its simplicity and reliability. The latest advancement in this area is the empirical scoring system (ESS), which Nelson standardized and adapted from prior models and empirical evidence [9].

The NSS evaluates physiological responses to relevant questions—those directly about the incident under investigation—against responses to comparison questions posed before and after the relevant ones. Responses are evaluated by either a 7-point or 3-point scoring system, based on the reaction times of 10, 15, or 20 s. Specifically, in the Utah technique, a positive score is assigned if the response to the comparison question exceeds that to the relevant question, a negative score if it is less, and a zero score otherwise. Each relevant question receives a score, and the aggregate of these scores determines the final decision [10].

An NSS uses physiological signals tied to deception as measurable indicators. These signals encompass blood pressure, respiration, and skin conductance, with primary features (physiological features) showing stronger links to deception than secondary features; therefore, secondary features might not be considered in certain models. ESSs and CSSs focus solely on primary features [4,5].

### 1.4. Computerized Scoring Systems

In polygraph tests, examiners analyze physiological responses to detect deception, typically using numerical scoring systems. However, examiners’ judgments can be influenced by their political, regional, and religious biases, as well as by personal factors such as fatigue and stress, potentially affecting the accuracy of the test results [11]. To solve these problems, computerized scoring systems (CSSs) have been developed to automatically analyze charts through software, aiming to reduce judgment errors. Extensive research has been conducted on CSSs, including the development of several commercial systems [12].

Notable examples of conventional CSSs include PolyScore, developed by the Johns Hopkins University Applied Physics Laboratory LLC in the USA, and CPS, from the University of Utah Psychology Laboratory in the USA. Both systems engage in signal processing, feature extraction, feature selection, and classification using statistical models. During signal processing, motion artifacts from movements and deep breathing are detected and removed, followed by the application of signal processing techniques. Features are then extracted from the bio-signals and standardized to compare the responses to relevant and comparison questions. The feature selection process reduces the dimensions of these features, which are subsequently used in the classification model to detect deception. PolyScore employs logistic regression for its classification model, whereas CPS utilizes Naïve Bayesian probability to calculate scores based on a linear combination of weighted features [3].

Five conventional algorithms, PolyScore, CPS, AXCON, Chart Analysis, and Identifi, demonstrated limited performance in a previous study utilizing data from 97 chart sets collected from criminal cases [12]. Of these, 56 chart sets were classified under the deception group and 41 under the non-deception group. Notably, the specificity of these algorithms was particularly low.

The American Polygraph Association conducted tests on over 250 students, finding near-perfect accuracy rates. However, it was cautious to claim absolute accuracy. Recent research suggests that the accuracy of new computerized polygraph systems approach 100%. UK-based studies have reported a 90% accuracy rate for detecting lies and a 70% rate for identifying truth through lie detectors. Lancaster University’s application of these tests on 180 students and staff revealed an accuracy close to 97%, albeit not perfect. Anderson et al. presented the results from polygraph brain mapping, achieving a 76% accuracy rate, while research from Britain and the Netherlands pointed to an 86% success rate. Despite these findings, many European countries remain skeptical of polygraph tests as reliable evidence, concluding the tests to be predominantly accurate but not infallible, with a reported 90% accuracy rate, suggesting their potential utility in investigative procedures [13].

Conventional algorithms, employing a linear classifier with a significant emphasis on skin conductance, exhibit limitations [14,15]. PolyScore and CPS use linear logistic regression and linear discriminant analysis, respectively, for deception detection [3]. The linear classifier benefits from straightforward computational processes and the ability to gauge the predictive variable’s contribution. However, its effectiveness is compromised by the nonlinear nature of bio-signals, which are the primary predictive variables in these cases [16].

### 1.5. Purpose

Polygraph examiners use physiological measurements and a scoring system to detect deception. Biases and personal factors can affect the results [11]. CSSs automate scoring and can be further enhanced by integrating deep learning structures such as deep neural networks (DNNs), to account for the nonlinearity of bio-signals.

This paper aims to develop a new polygraph system and a CSS that employs a deep learning classifier for automated deception detection.

## 2. Data Acquisition

In the previous section, we described the development of a polygraph device for measuring various bio-signals. To validate this device and develop a computerized scoring algorithm leveraging deep learning technology, it is necessary to gather a diverse set of data.

This section outlines the data collection methods and the results of experiments conducted using the newly developed polygraph device.

### 2.1. Research Direction

Traditional computerized scoring algorithms use linear classifiers, prioritizing skin conductance [14,15]. These classifiers offer the advantage of assigning weights to predictive variables and simplifying calculations. However, their reliance on linear relationships between variables reduces their accuracy when analyzing bio-signals, which exhibit nonlinear characteristics [16]. This can be overcome by integrating deep learning structures such as DNN to accommodate the nonlinearity of bio-signals.

For the development of a deep-learning-based scoring algorithm, it is imperative to collect adequate training and testing data. In this study, participants were classified into deception and non-deception groups. Their bio-signals were recorded during a polygraph test conducted after engaging in simulated scenarios and the Utah technique was employed, which is known for its high accuracy in Korea [17].

Participants were thoroughly briefed about the experimental procedure, potential risks, and the study’s objectives through a “Study Participant Information Sheet and Consent Form”, approved by the Institutional Review Board of the National Forensic Service (approval number: 906-180118-HR-003-02) and Wonju College, Yonsei University (approval number: 1041849-201803-BM-016-01). All participants provided written consent.

### 2.2. Participants

The study involved physically and mentally healthy adults who volunteered for the experiment, recruited via university announcements and online postings. They were informed about the experiment’s details, duration, compensation, and their rights over the phone. The participants were assured that their data would be discarded should they choose to withdraw from the study. The experiment had 92 participants, and the demographic information is presented in Table 1 and Table 2.

### 2.3. Research Instruments

Table 3 lists the instruments used in the polygraph tests.

### 2.4. Experimental Procedure

The experiment was designed and conducted in the Division of Forensic Psychology, Department of Forensic Medicine, at the National Forensic Service (NFS). Participants were asked to select and enact one of two scenarios, either asserting truths or concealing lies related to the scenario. The experiment incentivized active participation by offering maximum compensation for successfully demonstrating truthfulness or the concealment of lies, with reduced compensation for other outcomes.

Scenario 1: Conditions under which a crime is carried out

1. The participant reads and understands the instructions provided in the envelope.
2. Carrying the selected instructions in their bag alongside another set of instructions, the participant can review the selected instructions if required.
3. Upon entering the office to enact the scenario, if someone is present, the participant introduces themselves as the person assigned to clean the office.
4. Should the office be empty or the person temporarily leaves, the participant proceeds to clean the desk.
5. During the task, the participant discovers a ring within a desk drawer.
6. Subtly, the participant takes the ring, conceals it among their belongings, and leaves the room secretly.
7. The participant continues these tasks undetected until the office’s occupant returns, ensuring their actions remain undiscovered.
8. After leaving the office, the participants wait in the waiting area where interviews and a polygraph test about the supposed theft of the ring are conducted.

Here, the participant must consistently deny any knowledge of the ring’s theft or possession.

Scenario 2: Conditions under which a crime is not carried out

1. The participant ensures they are fully aware of the instructions within the envelope.
2. They keep the selected instructions in their bag, along with another set of instructions, for reference if needed.
3. Upon executing the scenario in the office, the participant introduces themselves to anyone present as the cleaner.
4. In the absence of others or if left alone, the participant cleans the office desk.
5. Following their departure from the office and while in the waiting area, interviews and a polygraph test are conducted concerning the alleged theft of the ring. The participant is to deny any knowledge of whether the ring was stolen or to confirm it was not stolen by them.

### 2.5. Experimental Data

The experiment involved 91 participants, and 78 datasets were used for the analysis after excluding 13 datasets due to strong motion artifacts.

## 3. Deception Detection Algorithm

This section builds on the previous discussions on data acquisition using the polygraph device developed in Section 2. It introduces the creation of a computerized scoring algorithm using DNN to account for the nonlinearity of bio-signals. Specifically, the algorithm employs convolutional neural networks (CNNs) [18] and recurrent neural networks (RNNs) [19].

### 3.1. Deep Learning Architecture for Deception Detection

**Figure 1:**
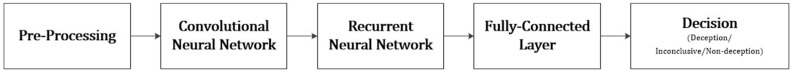
Overall structure of the deception detection algorithm.

In this study, we design a deep-learning-based algorithm for detecting deception (Figure 1). The algorithm processes input bio-signals including photoplethysmogram (PPG), blood pressure, thoracic and abdominal respiration, and skin conductance, classifying the output as either deceptive or non-deceptive based on the detection results.

Initially, the input data are compressed through a CNN structure, after which the compressed time-series data are further processed using an RNN structure. A fully connected layer then classifies the results as either truthful or deceptive. The cumulative results from the fully connected layer for each question form a single series outcome, ultimately categorizing participants into deceptive or non-deceptive groups.

**Figure 2:**
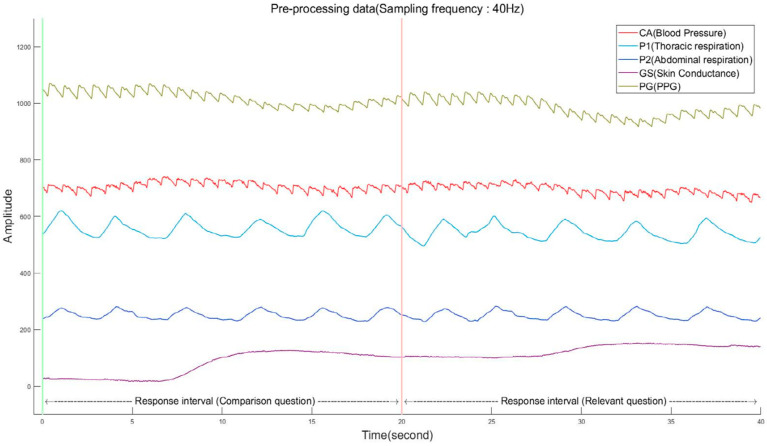
Pre-processing results of the input bio-signals (sampling frequency: 40 Hz).

Figure 2 highlights how the algorithm addresses discontinuities at the boundary between bio-signals responding to comparison and relevant questions. When concatenating two signals, removing discontinuities is simply achieved by adding an offset to the second signal to concatenate the last data point of the first signal with the first data point of the second signal.

#### 3.1.1. CNN

**Figure 3:**
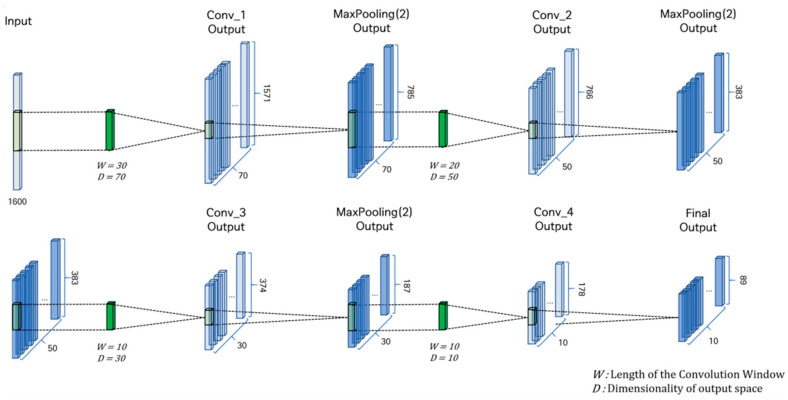
Convolutional layer of the deep learning architecture for deception detection.

A specialized CNN architecture compresses the measured bio-signals, including blood pressure, thoracic and abdominal respiration, skin conductance, and PPG (Figure 3). This CNN consists of four convolutional layers, each employing a weight filter initialized with a random noise matrix. The CNN comprises four layers, each with a distinct number of filters: 70, 50, 30, and 10 in layers 1–4, respectively. Correspondingly, the filter sizes are set at 70, 50, 30, and 10 for layers 1–4, respectively. The rectified linear unit (ReLU) serves as the activation function across all layers. To identify the significant features, max pooling is applied within the pooling layer, employing a kernel size of two. To mitigate overfitting due to the extensive input vectors in each convolutional layer, dropout rates of 50% are applied to the first and second layers, and a 25% dropout rate is applied to the third and fourth layers.

#### 3.1.2. RNN

**Figure 4:**
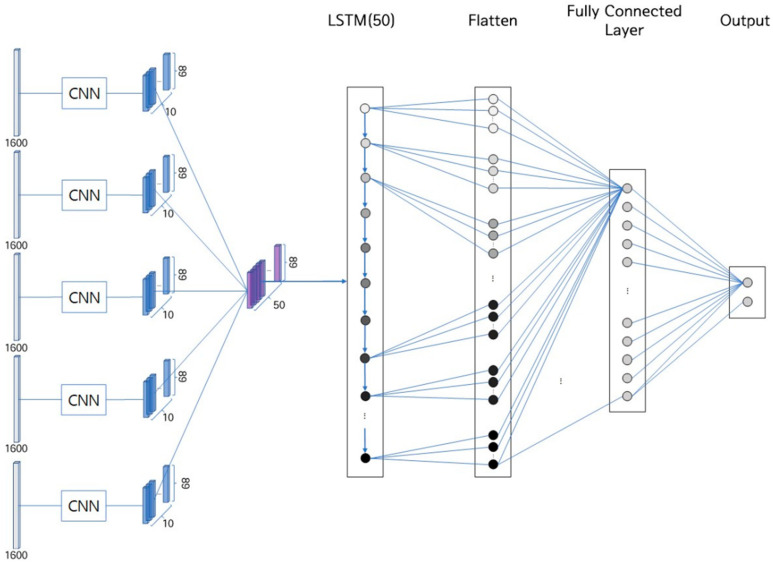
The CNN, LSTM, and fully connected layer of the deep learning architecture for deception detection.

The compressed bio-signals, now in a 1-D time-series format after CNN processing, are further analyzed using an RNN structure (Figure 4). The CNN-compressed bio-signals are merged into a single output through a merge layer before being fed into the RNN, which consists of a single long short-term memory (LSTM) layer. This LSTM layer is configured to output all elements for each input, utilizing a hyperbolic tangent as the activation function. The LSTM’s output—an 89 × 50 matrix, equivalent to 4450 1-D matrices—is flattened and introduced into a fully connected layer for the final classification process.

#### 3.1.3. Fully Connected Layer

The fully connected layer (Figure 4) plays a crucial role in classifying the responses to questions as either true or false, based on the signals processed by the RNN. It consists of two layers: the first uses the ReLU function for activation, setting the output size to 50, while the second layer uses the Softmax function for activation, assigning an output size of two. This configuration enables the output to be presented as a probability value, indicating the likelihood of the responses being false or true.

#### 3.1.4. Classification of Deception Detection

**Figure 5:**
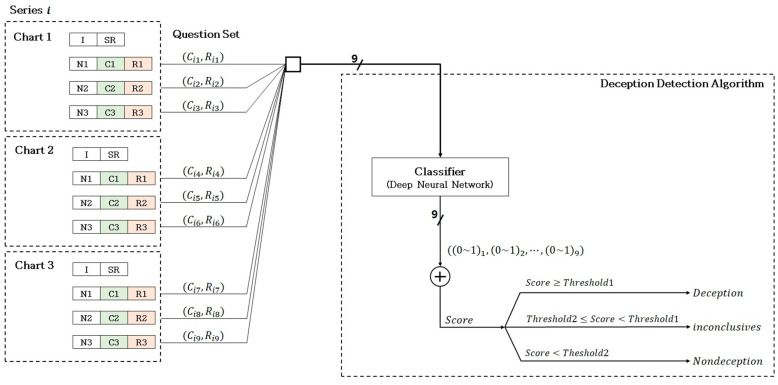
Process of deception detection using the developed algorithm.

Utilizing the deep learning architecture outlined previously, this algorithm classifies participants into deceptive or non-deceptive groups. The model, once trained, classifies experimental data and aggregates the results for each question series per participant to detect deceptive behavior. Participants who perform Scenario 1 produce one series of truthful responses and one of the false responses, whereas participants in Scenario 2 generate two series of truthful responses. Each series involves three charts, with each chart encompassing three sets of questions, including both comparison and relevant questions. Figure 5 shows the methodology for measuring three charts per series and employing three question sets per chart to detect deception. The classifier assigns a probability value ranging from 0 to 1 for each question set, categorizing them as true or false, and calculates the score by summing these results. A score above the threshold of one indicates deception, while a score below two signifies non-deception. Scores falling between these thresholds are deemed inconclusive.

### 3.2. Experimental Results

To assess the performance of the developed algorithm, we compared its scoring results with those from the PolyScore (Lafayette Instrument Company, Lafayette, IN, USA) and OSS-3 [8] established CSSs. We employed K-fold cross-validation for performance evaluation, calculating recall, precision, and F1 score to quantitatively evaluate classification accuracy. Recall measures the algorithm’s ability to correctly identify deceptive groups, while precision assesses its success in accurately classifying non-deceptive instances.

#### 3.2.1. Data Classification

This study analyzed a total of 702 experimental data points, including 378 false and 324 true data points. The discrepancy in the number of false and true data points led to varied performance outcomes based on the classification of training and test data. Therefore, repeated training and testing through cross-validation were conducted on the datasets to determine the mean performance and variance in performance metrics.

K-fold cross-validation is a method for verifying the robustness of a dataset by dividing it into K subsets. In this process, one subset is used as the test data, while the remaining K-1 subsets serve as the training data. This procedure is repeated such that each subset serves as the test data once, enhancing the reliability of the performance evaluation by mitigating the influence of any anomalies present in a limited dataset [20]. In this study, we employed a 10-fold cross-validation method, maintaining a training-to-test data ratio of 9:1.

The algorithm developed for this study utilized a deep neural network model, as described in Section 3.1, to estimate the probability of a question set—comprising one comparison question and one relevant question—being false, ranging from 0 to 1. The model’s cost function was defined by the Softmax cross-entropy. This function calculates the normalized value for the cross-entropy, averaging it over the outputs processed by the Softmax function in the output layer. The optimization of the model was achieved through the Adam function, which combines the advantages of both the Adadelta and RMSprop algorithms. It considers both the moving average of the gradients and their square values, setting the learning rate at 0.0015 and the batch size at 64.

#### 3.2.2. Deception Detection

**Figure 6:**
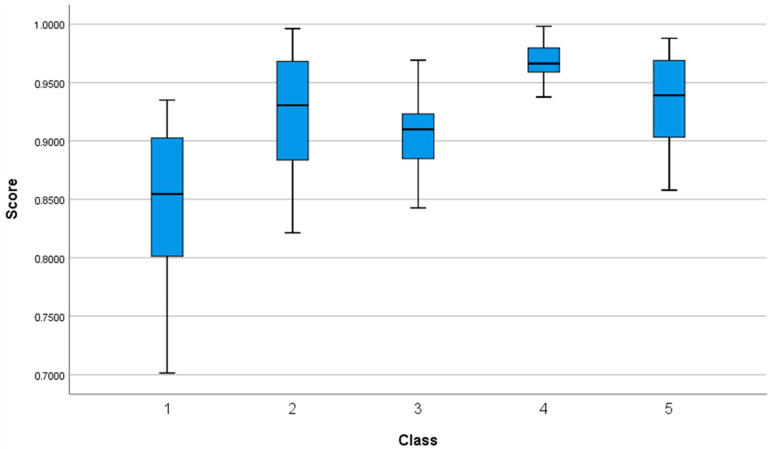
Boxplot of classification results of 10-fold cross-validation.

The classification results of the question sets, analyzed using the developed algorithm with 10-fold cross-validation, are shown in Figure 6 and Table 4.

These results were statistically examined using the IBM SPSS 23 Statistics software. Given that there were ten samples for each DNN structure, a normality test was performed, confirming that all results from the 10-fold cross-validation for each DNN structure met the normality criteria at a 0.05 significance level (Table 5).

In this study, multiple samples were collected from various participants, and since the model evaluated each sample individually, the assumption of independence was not entirely violated. Furthermore, by utilizing repeated measures ANOVA or mixed-effects models, it was possible to account for correlations between samples while quantitatively analyzing differences between groups. Therefore, the use of ANOVA in this study did not undermine the validity of the research.

By contrast, the one-way repeated analysis of variance (ANOVA) showed no significant difference across the structures, except for structure 1, at the 0.05 significance level (Table 6).

Structure 4, which incorporates five CNNs with four layers and one LSTM, emerged as the superior DNN structure based on its highest mean and lowest standard deviation in its F1 scores (Table 4).

Each series comprised nine categorized question sets, with the aggregated score from these sets ranging from a maximum of 9 (indicating all responses were deceptive) to a minimum of 0 (indicating all responses were truthful). A score above one categorized a participant into the deception group, while a score below two placed them in the non-deception group. Scores falling between *threshold 1* and *threshold 2* (defined using Equations (1) and (2), respectfully) deemed the outcome inconclusive. These thresholds were derived using the Utah scoring method, where each question contributed ±2 points, leading to a total score range of –18 to +18. A score above +5 indicated deception, below –5 indicated non-deception, and scores within –5 to +5 were inconclusive. To adapt this method for our algorithm, we defined *threshold 1* and *threshold 2*, respectively, using Equations (1) and (2) [21]:

*Threshold* 1 = ((+6) + 18)/(18 − (−18)) × (*score*_max_ − *score*_min_) = 6 (1)

*Threshold* 2 = ((−6) + 18)/(18 − (−18)) × (score_max_ − score_min_) = 3 (2)

where *score*_max_ and *score*_min_ denote the maximum and minimum values of the score, respectively.

The probability of deception for each question was calculated by using the most effective classifier (Table 4). The algorithm then categorized each participant into either the deception or non-deception group based on the cumulative probabilities of the nine questions. Table 7 shows the classification results for 78 participants, with “Correct” denoting an accurate classification, “Incorrect” for misclassifications, and “Inconclusive” for undetermined cases.

#### 3.2.3. Comparison with Conventional Algorithms

To assess the performance of the algorithm, we compared its scoring results with those from the conventional computerized scoring algorithms PolyScore and OSS-3 [8]. Table 8 presents the comparative analysis results. Additionally, to evaluate the importance of incorporating the PPG signal, which reflects the cardiac activity alongside the blood pressure signal but is not measured in conventional polygraphs, the algorithm’s performance was evaluated with and without the PPG signal.

## 4. Discussion

This research aimed to develop a Korean polygraph system, leveraging multichannel signals for effective criminal investigation and legal evidence, as a replacement for imported polygraph systems not optimized for Korea’s investigative environment. Furthermore, a Korean CSS was devised, utilizing a DNN to minimize the subjective biases of polygraph examiners and improve accuracy by accounting for the nonlinearity of bio-signals.

The polygraph developed in this study was equipped to measure blood pressure, thoracic respiration, abdominal respiration, skin conductance, and PPG. It interfaces with PC software via USB to transmit the bio-signals measured during tests. The software facilitated file management, questionnaire editing, graph review, and printing functions. It enabled the real-time observation of participant responses throughout the examination. Post-test, the examiner analyzed the data to detect deception.

The computerized scoring algorithm processed five types of input signals from the polygraph to automatically detect deception. It featured a structure that integrated five parallel-merged layers, including a CNN and an RNN, culminating in a fully connected layer. Deception detection within a series—comprising nine question sets—relied on the cumulative score from these sets. A score above one signified deception, below two indicated non-deception, and scores within these thresholds were deemed inconclusive.

Algorithm performance was assessed through recall, precision, and accuracy metrics, using 10-fold cross-validation on the data from 78 participants, which included 42 deceptive and 36 non-deceptive series, totaling 702 data points. The evaluation of the DNN structure, incorporating five four-layer CNNs and one LSTM unit, yielded a recall of 0.9681 ± 0.0314, precision of 0.9700 ± 0.0321, and F1 score of 0.9683 ± 0.0171. By contrast, the DNN structure with only five four-layer CNNs showed a recall of 0.9314 ± 0.0473, precision of 0.9377 ± 0.0529, and F1 score of 0.9340 ± 0.0449, underscoring the critical role of LSTM in enhancing performance.

The algorithm’s performance was compared with the PolyScore and OSS-3 established algorithms. In tests comprising 42 deception series and 36 non-deception series, PolyScore discriminated 17 deception series correctly, but misclassified 6 and labeled 19 as inconclusive. It correctly identified 15 non-deception series, misclassified 5 as deceptive, and deemed 16 inconclusive. OSS-3 discriminated 24 deception series, misclassified 18, and had no inconclusive outcomes, while correctly identifying 28 non-deception series, misclassifying 7 as deception, and marking 1 as inconclusive. The developed algorithm, excluding the PPG signal, discriminated 36 out of 42 deception series, with none misclassified and 5 inconclusive. It correctly identified 35 out of 36 non-deception series, with none misclassified and 1 inconclusive. With the inclusion of the PPG signal, the algorithm discriminated 41 out of 42 deception series, with none misclassified and only 1 inconclusive, and it correctly identified all non-deception series. Therefore, the developed algorithm outperformed the conventional computer scoring algorithms, and the inclusion of the PPG signal, which reflects cardiac activity, further enhanced its performance compared to the algorithm without the PPG signal.

The superior performance of the developed algorithm, especially with the PPG signal, was attributed to the ability of its CNNs to process nonlinearity and the capacity of its RNNs for handling time-series data, enabling effective signal compression, feature extraction, and classification. This contrasts with conventional algorithms that rely on linear classifiers. The relatively lower recall rate compared to precision and accuracy may be due to the disproportionate number of deception series data versus non-deception series data, indicating the need for a larger dataset to further improve the algorithm’s performance.

## 5. Conclusions

Polygraph devices that measure blood pressure, respiration, and skin conductance are used as legal evidence in criminal investigations in many countries. Korea introduced its first polygraph device in 1960, but has relied on imports due to the absence of domestic development.

To detect deception in a polygraph test, examiners analyze physiological data on a chart, often using a numerical system. However, biases such as political or religious views can lead to errors. Fatigue and stress may also affect the results [11]. Introducing CSSs automates chart scoring to reduce mistakes, and integrating deep learning structures such as DNNs can enhance performance.

Accordingly, this study developed a Korean polygraph designed for effective criminal investigations and legal evidence, tailored to Korea’s specific needs and reducing reliance on imported devices. A Korean CSS, leveraging a DNN, was also developed to mitigate the subjective biases of examiners and to enhance accuracy by considering bio-signal nonlinearity.

Data were acquired using the newly developed polygraph, and a deep-learning-based computerized scoring algorithm was developed, outperforming conventional algorithms such as PolyScore and OSS-3.

This study had several limitations. To enhance and stabilize these scoring algorithms, the training dataset should be expanded. Connecting the developed polygraph to a central server via Ethernet and distributing it across investigation agencies can significantly increase the data collection and training data volume. Integrating the developed algorithm directly into the polygraph software could support examiners with real-time evaluations.

Future research should focus on constructing a Korean computerized scoring network by connecting the developed polygraph devices to a central server. This network would manage the training data collected from distributed polygraphs, facilitating a comprehensive and long-term study on improving the algorithm’s performance through periodic training with new data. The data used in this study were obtained from subjects commissioned for criminal investigations; therefore, the amount of data was limited and further experiments could not be conducted. Future studies should be conducted using a larger number of subjects.

## Figures and Tables

**Table 1:** Number of participants by group/gender.

	Non-Deception	Group	Deception	Group
By group	46		46	
By gender	Female	Male	Female	Male
	29	17	33	13

**Table 2:** Ages of non-deception/deception groups.

Number of Participants				Mean	Standard Deviation	Median	Min	Max
Non-deception	46	Male	17	22.88	2.47	23	19	27
		Female	29	20.86	1.3	21	19	25
	45	Male	13	23.38	1.98	23	20	27
Deception	(excluding 1)	Female	32 (excluding 1)	20.38	0.94	21	19	23

**Table 3:** Research instruments and their use.

Instruments	Model	Use
Polygraph device	Made for this research	To measure bio-signals during the polygraph test
	LX5000	For comparison with the polygraph device developed in this study
PC software	Made for this research	To display and save bio-signals during the polygraph test
Webcam	HD Pro Webcam C920	To record the participant during the polygraph test

**Table 4:** Classification results of 10-fold cross-validation (mean ± SD).

No.	DNN Structure	Recall	Precision	F1 Score	Computation Time (s)
1	5 CNNs (1-layer) + 1 LSTM	0.8440 ± 0.1286	0.8632 ± 0.0807	0.8433 ± 0.0731	3514
2	5 CNNs (2-layer) + 1 LSTM	0.9201 ± 0.0568	0.9330 ± 0.0849	0.9239 ± 0.0551	2324
3	5 CNNs (3-layer) + 1 LSTM	0.9256 ± 0.0644	0.8920 ± 0.0461	0.9062 ± 0.0344	1862
**4**	**5 CNNs (4-layer) + 1 LSTM**	**0.9681 ± 0.0314**	**0.9700 ± 0.0321**	**0.9683 ± 0.0171**	**1273**
5	5 CNNs (4-layer) only	0.9314 ± 0.0473	0.9377 ± 0.0529	0.9340 ± 0.0449	891

**Table 5:** Results of the normality test for the results of 10-fold cross-validation of five DNN structures.

		Kolmogorov–Smirnov			Shapiro–Wilk		
	No.	Statistic	DoF	*p* -Value	Statistic	DoF	*p* -Value
F1 score	1	0.193	10	0.200	0.932	10	0.471
	2	0.129	10	0.200	0.945	10	0.607
	3	0.179	10	0.200	0.954	10	0.714
	4	0.197	10	0.200	0.930	10	0.445
	5	0.110	10	0.200	0.973	10	0.916

**Table 6:** Results of one-way repeated ANOVA tests for each DNN structure.

						95% Confidence Interval	
	(I) Class	(J) Class	Difference (I–J)	Standard Error	*p*-Value	Lower Limit	Upper Limit
Tukey HSD	4	1	0.1250400	0.0218870	0.000	0.062849	0.187231
		2	0.0444200	0.0218870	00.269	−0.017771	0.106611
		3	0.0620800	0.0218870	0.051	−0.000111	0.124271
		5	0.0343200	0.0218870	0.525	−0.027871	0.096511
Bonferroni	4	1	0.1250400	0.0218870	0.000	0.060428	0.189652
		2	0.0444200	0.0218870	0.483	−0.020192	0.109032
		3	0.0620800	0.0218870	0.068	−0.002532	0.126692
		5	0.0343200	0.0218870	1.000	−0.030292	0.098932
Dunnett T3	4	1	0.1250400	0.0239032	0.003	0.042172	0.207908
		2	0.0444200	0.0184034	0.249	−0.018431	0.107271
		3	0.0620800	0.0122859	0.002	0.021680	0.102480
		5	0.0343200	0.0150918	0.299	−0.016390	0.085030

**Table 7:** Classification results of the deception and non-deception groups.

Deception Series (N = 42)			Non-Deception Series (N = 36)		
Correct	Incorrect	Inconclusive	Correct	Incorrect	Inconclusive
41	0	1	36	0	0

**Table 8:** Comparison with conventional algorithms.

	Deception Series (N = 42)			Non-Deception Series (N = 36)		
	Correct	Incorrect	Inconclusive	Correct	Incorrect	Inconclusive
PolyScore	17	6	19	15	5	16
OSS-3	24	18	0	28	7	1
This study (w/o PPG)	37	0	5	35	0	1
**This study (w/PPG)**	**41**	**0**	**1**	**36**	**0**	**0**

## Data Availability

The data that support the findings of this study can be made available upon reasonable request to the corresponding author.

## Abbreviations

The following abbreviations are used in this manuscript:

ASTM	American Society for Testing and Materials
CIT	Concealed Information Test
CNN	Convolutional Neural Network
CPS	Computerized Polygraph Scoring System
CQT	Comparison Question Test
CSS	Computerized Scoring System
ESS	Empirical Scoring System
LSTM	Long Short-Term Memory
NFS	National Forensic Service
NSS	Numerical Scoring System
PPG	Photoplethysmogram
ReLU	Rectified Linear Unit
RNN	Recurrent Neural Network
